# How Robust Is Your Project? From Local Failures to Global Catastrophes: A Complex Networks Approach to Project Systemic Risk

**DOI:** 10.1371/journal.pone.0142469

**Published:** 2015-11-25

**Authors:** Christos Ellinas, Neil Allan, Christopher Durugbo, Anders Johansson

**Affiliations:** 1 Systems IDC, University of Bristol, Bristol, United Kingdom; 2 Systemic Consult Ltd, Bradford-on-Avon, United Kingdom; 3 School of Management, University of Bath, Bath, United Kingdom; 4 Management School, University of Liverpool, Liverpool, United Kingdom; Beihang University, CHINA

## Abstract

Current societal requirements necessitate the effective delivery of complex projects that can do more while using less. Yet, recent large-scale project failures suggest that our ability to successfully deliver them is still at its infancy. Such failures can be seen to arise through various failure mechanisms; this work focuses on one such mechanism. Specifically, it examines the likelihood of a project sustaining a large-scale catastrophe, as triggered by single task failure and delivered via a cascading process. To do so, an analytical model was developed and tested on an empirical dataset by the means of numerical simulation. This paper makes three main contributions. First, it provides a methodology to identify the tasks most capable of impacting a project. In doing so, it is noted that a significant number of tasks induce no cascades, while a handful are capable of triggering surprisingly large ones. Secondly, it illustrates that crude task characteristics cannot aid in identifying them, highlighting the complexity of the underlying process and the utility of this approach. Thirdly, it draws parallels with systems encountered within the natural sciences by noting the emergence of self-organised criticality, commonly found within natural systems. These findings strengthen the need to account for structural intricacies of a project’s underlying task precedence structure as they can provide the conditions upon which large-scale catastrophes materialise.

## Introduction

Improved planning and resource allocation is an intricate part of decision making. Within a project context, task decomposition and dependency mapping are usual ways of improving it. Standard practice for doing so, for modelling any kind of project, relies on the use of task precedence data [1]. Currently, the majority of tools adopt an activity-on-arc network representation [2], building on concepts first introduced by the Critical Path Method [3] and Program Evaluation and Review Technique [4]. This view is formally constructed using temporal elements of the project (e.g. task duration as the length of the arc). As such, the main emphasis is on the temporal aspects of Project Management (PM) e.g. improving the estimation of the project’s overall duration, while acknowledging the uncertain duration of its composing tasks [2, 5–7]. Approaches of this sort do not focus on the non-trivial topology of the underlying precedence network. As a consequence, a number of fundamental deficiencies arises–Williams [8] categorises them as follows:

- *Feedback loops*: Consider a task that is conditional on some sort of quality test. Failure to comply will immediately lead to the task being repeated. Though important, our work will not focus on this aspect;
- *Non-linear relationships*: Cause and effect are not necessarily proportionate;
- *Emergent behaviours*: Complete predictability of the behaviour of a system at one scale does not necessarily imply complete predictability of the system at a different scale.

Emergence is a fundamental property of complex systems [9, 10]—understanding its impact on systemic performance is of great interest as undesirable, systemic effects (e.g. global catastrophe fuelled by local failures) have been linked to the loss of billions of USD and, in some cases, human life [11–13]. Managing these systemic effects has been a challenging task across numerous diverse fields, ranging from epidemiology [14–17] and finance [18, 19] to infrastructure management [20, 21]. Despite the contextual differences of these domains, the underlying approach for understanding these systemic effects tends to be similar. Specifically, mathematical models are used to define the *process* that drives these systemic effects, and numerical simulation to assess its validity and practical relevance [22]. Complex networks have proved to be a highly successful framework in sustaining this approach, adequately capturing the intricacies (in the form of non-linear interactions) that underlie the *structure* of these systems [23–26]. Under this view, such catastrophes are seen as the emergent outcome of a sequential progression of failures (or *a cascade*) as they propagate throughout the system’s structure (or *network*) [22, 26]. One way of modelling these cascades is through the use of a threshold model, first introduced in [27] to explain social segregation. Variants of this model have been subsequently applied to a wide range of contexts, ranging from social contagion [15, 28] and disease propagation [14, 16, 17] to systemic risk [19, 29, 30] suggesting its capacity to serve as a unifying framework for modelling similar systemic effects [18].

By adopting a similar approach, the likelihood of a project sustaining large scale catastrophes, as triggered by local failures, can be assessed. Though insight of this sort may uncover numerous interesting *statistical* properties of the system [26], they are of little practical use to a project manager. This incompatibility arises from the nature of control that a project manager can exert on the system i.e. he/she can only manage the task(s) that are currently active. In other words, global information, such as statistical descriptions, is of little practical use if the project manager is limited to local action [31]. As such, the industry is slowly transitioning from the once-ubiquitous notion of a critical path (global information) to individual measures of task criticality (local information) [32]. In the context of cascading failures, such local information revolves around identifying the nodes responsible for their emergence, forming the main research question:

*Can we quantitatively assess the likely impact that a single, local failure in a project will have at a global level?*

To evaluate the research question, an analytical model is devised and subsequently tested on an empirical dataset using numerical simulation. Typically, means of this sort tend be both time consuming and resource intensive. This is in contrast with regular decisions making, which takes place within the restrictions of limited budget and stringent timeframes. To reflect this reality, the capacity of task information to serve as proxies for identifying these critical tasks will also be explored.

This work makes three contributions. Firstly, proposes a model that can replicate a cascading process across any type of project while using widely-captured and readily-available data. Similar approaches have been used to explore the robustness of numerous complex systems [26], yet project robustness has eluded a similar treatment—this work aims to fill this gap. By doing so, the capacity of each task to trigger a catastrophe can be quantitatively assessed. Importantly, this work highlights that both local and global failures can arise from the exact same mechanism–insights of this sort are in agreement with similar observations from numerous other systems [33]. Secondly, this work shows that simple task information is incapable of serving as a proxy for identifying these critical tasks–further analysis shows similar inefficiencies in using network-based centrality measures (i.e. degree, betweenness). Thirdly, it provides analogies between project behaviour and natural systems, hinting a link with a theory describing the behaviour of the latter. By doing so, it hints that general theories with wide (or even universal) project applicability may be possible. This is a widely sought but currently eluding quality for management science [34] as project uniqueness is deeply engrained within the literature [1], and contradicts the very ethos of a project-focused theory.

The remaining part of the paper will be structured as follows: the Data section will introduce the dataset used. The Model section will briefly introduce the concept of the model, followed by formal definitions of its key components, along with the algorithm used to deliver the dynamic. The Result section contains the initial configuration of the model and the subsequent results of the analysis. A Discussion section will provide the interpretation of the results, followed by a Conclusion section. Supplementary Information provides further supporting evidence for this work, including the raw file (and derived dataset) used, a worked example of the algorithm and further results.

## Data

Engineering projects with a focus on technical activities form a representative class of modern organisation activity [34]—construction projects being a subset of them. As such, empirical task precedence information (in the form of a Gantt chart) of a construction project were obtained and used to construct an activity-on-node (AON) network. The aim of the project was to deliver a Commercial Office complex, over a period of 743 days (September 2004 to September 2006) with an agreed cost of approximately 13 million USD. The Gantt chart was last modified 603 days after the project was started.

**Fig 1:**
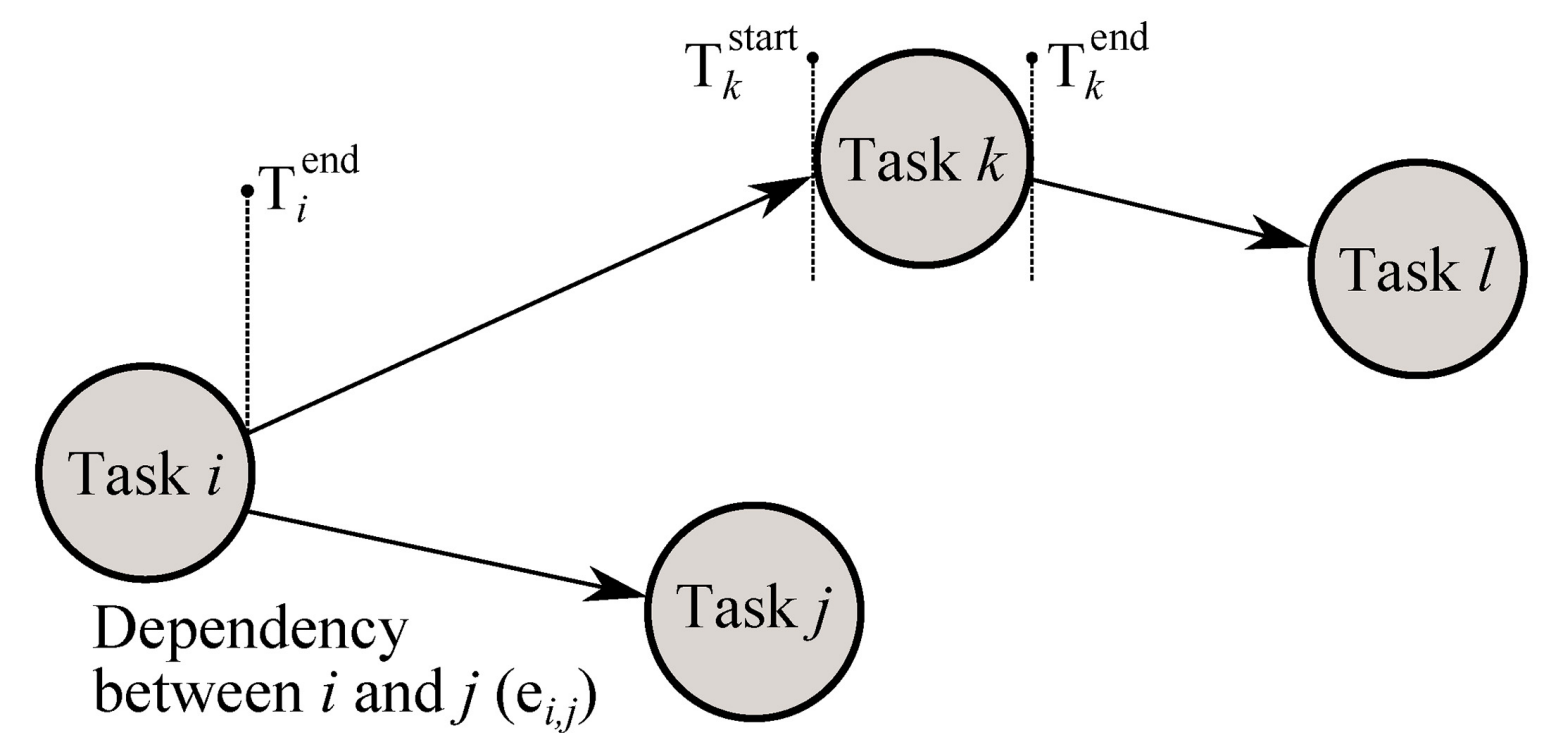
AON network abstraction. Example project illustrating the network view adopted throughout this work.

**Fig 2:**
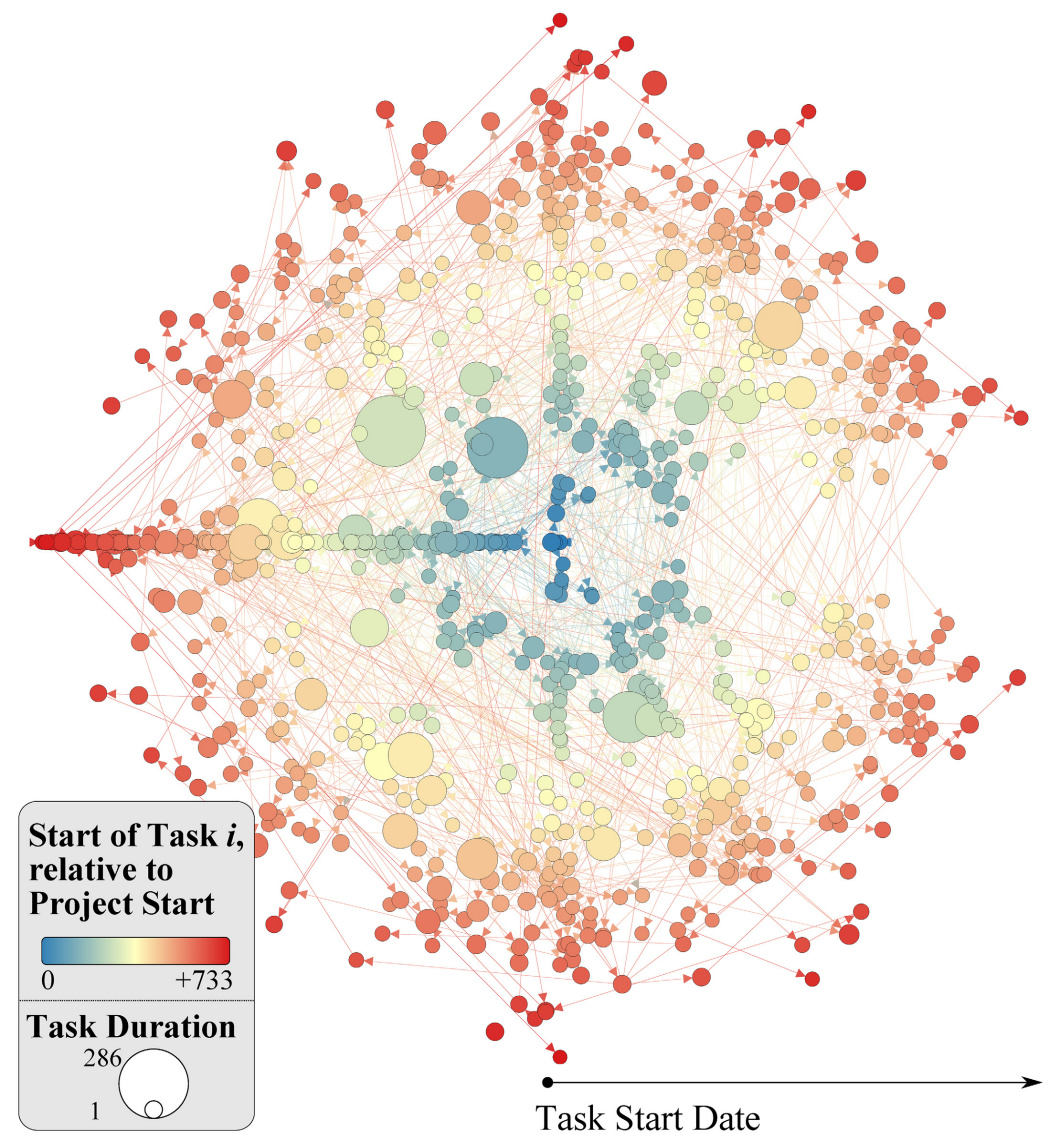
AON network of the empirical dataset. Each node corresponds to a task; each directed link to a precedence relationship. Node size corresponds to task duration; node colour and position corresponds to a task’s start data, relative to the start of the project (e.g. darker and further away from centre indicates a task which started at a later stage).

Under an activity-on-node (AON) network, a project is abstracted as a directed graph *G* = {{*N*} {*E*}}, in which every task *i* is abstracted as node *i*, *i* ∈ *N*. Similarly, a dependency between task *i* and *j* is mapped using a directed link (or *edge*) *e*_*i*,*j*_, where *e*_*i*,*j*_ ∈ *E*. It is worth noting that an AON network provides for a more general and flexible approach when compared to the traditional activity-on-arc network view, adequately capturing structural intricacies of the task precedence information [35, 36]. The start and end date of task *i* is noted as $T_{i}^{\text{start}}$ and $T_{i}^{\text{end}}$ respectively–see Fig 1 for a trivial example; Fig 2 for the actual AON network used, composed of 833 nodes and 806 edges. The original Gantt chart is provides in S1 Dataset; the resulting AON network in S1 Table; details of individual tasks are included in S2 Table; an overview of the method from converting a Gantt chart to the AON network can be found in S1 Text.

It should be noted that AON networks have a rather distinct character from other forms of networks, both in terms of topological features and the level of information that they capture. In terms of their topology, AON network are composed of distinct concentrations of 3-node subgraphs (or structural motifs [37]), distinguishing them from other natural and engineered systems [38]. In terms of information captured, AON networks include both global (i.e. topological features) and local information (i.e. task duration); along with an interpretation on the Euclidean space of a network (i.e. length of a link corresponds to the time between two consecutive tasks). The latter feature is reminiscent of spatial networks (where length of a link corresponds to physical distance). Yet, a number of assumptions that underlies spatial networks (e.g. link preservation comes at a cost [39], loops are allowed [40]) limits their commonalities. Similarly, synthetic networks, constructed using some form of generative process (e.g. preferential attachment [41], rewiring [42]), have few commonalities with AON networks. Specifically, such networks restrict themselves in replicating specific statistical features of a network’s topology (e.g. heavy-tail degree distribution; high clustering with low average path length). Though extremely useful, such models are bound to miss important information (i.e. task duration; time between consecutives tasks) whilst ignore various restrictions (i.e. absence of loops). We thus consider the construction of a generative mechanism that can capture all vital information whilst satisfying some distinct topological features to be an open challenge.

## Model

### Overview

The failure of an individual node can be described by considering a tipping point, where the state of a node changes if a threshold value is exceeded. Such a threshold value may be interpreted as the capacity of a node to operate and hence, must capture relevant contextual information. In the context of projects, one such quantity is the quality of completion of task *i*. The impact of task *i* failing can be explored numerically by artificially failing it (i.e. switch its state from “non-affected” to “affected”) and examining its capacity to affect its neighbouring task(s) *j*—these tasks are considered to be stressed. The amount of stress that they receive is a function of node’s *j* individual characteristics that can affect its exposure to this stress (i.e. its “sensitivity”), along with the efficiency at which the failure of task *i* is being conveyed (i.e. its “spreading power”). As a result of the combination of these intrinsic and extrinsic influences, the threshold of task *j* decreases. In relative terms, if this change is greater than a given percentage of its original threshold, task *j* fails and now has the opportunity to fuel the cascade further by affecting its own neighbours. Once the cascade process has been brought to an end (i.e. task *i* has no neighbours; neighbouring task(s) have resisted failure), the effect is recorded and the state of all tasks is reset to “non-affected”–this process is iterated across all tasks.

### Intrinsic Nodal Characteristics

**Fig 3:**
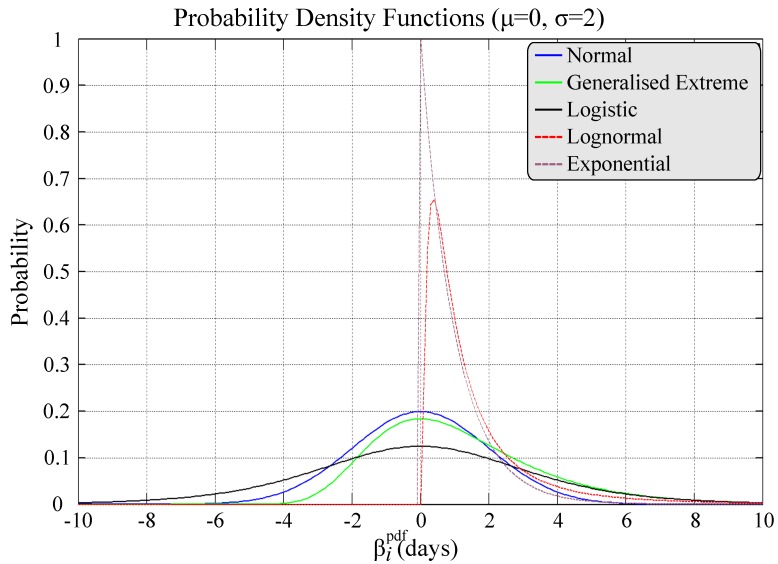
Example pdfs from which $\beta_{i}^{\text{pdf}}$ ensembles may be drawn from.

Links found within the AON network can be seen as functional dependences, where the start of task *j* is conditional to the successful delivery of all of its predecessors (i.e. task *i*). Consequently, the quality of completion of task *i* (*q*_*i*_) will inevitably influence the potential quality of task *j* i.e. the higher the former, the more probable the successful (i.e. high quality) delivery of the latter. Quality is a function of numerous parameters, one of them being the ability to dispense resources (e.g. time, material, labour, equipment etc.) as originally planned. Consequently, the ratio between the time resource, measured in days, assigned for completing task $i\;\left(T_{i}^{\text{plan}}\right)$ against the one actually delivered $\left(T_{i}^{\text{actual}}\right)$, defined as ${\hat{T}}_{i}=\frac{T_{i}^{\text{actual}}}{T_{i}^{\text{plan}}}$, can be used as an input for obtaining *q*_*i*_. The term $T_{i}^{\text{actual}}$ acknowledges the uncertain nature of the environment in which the project takes place. It is defined as

$$T_{i}^{\text{actual}}=\{\begin{array}{c} T_{i}^{\text{plan}}+\beta_{i}^{\text{pdf}},\;\text{if}\;T_{i}^{\text{plan}}+\beta_{i}^{\text{pdf}}>0\\ 1\;\text{otherwise} \end{array}$$ (1)

where $\beta_{i}^{\text{pdf}}$ serves as a perturbation term, effectively accounting for this uncertainty. Note that a conditional definition is required in order to avoid possible negative $T_{i}^{\text{actual}}$ values (e.g. when $\beta_{i}^{\text{pdf}}$ is negative and greater than $T_{i}^{\text{plan}}$). For each task *i*, an ensemble of 1,000 $\beta_{i}^{\text{pdf}}$ values is drawn from a given probability density function (pdf), see Fig 3, before suitably aggregated to a single value per task. Specifically, the 95^th^ percentile value of the ensemble of 1,000 $\beta_{i}^{\text{pdf}}$ values is used to derive a worst-case scenario for every $T_{i}^{\text{actual}}$. Clearly, the nature of the pdf will dictate both the variance and size of obtainable $\beta_{i}^{\text{pdf}}$ values, reflecting a relatively uncertain or stable environment. Within this work, the latter is simulated via the use of a Normal pdf i.e. $\beta_{i}^{\text{pdf}}∼N\left(0,2\right)$. We note that the influence of ${\hat{T}}_{i}$ is restricted in describing the initial quality of task *i*, defining its tipping point (see Eq 11). As such, the quality of completion of each task (as a function of ${\hat{T}}_{i}$), its spreading power and sensitivity provide an initial description for each task *i*, but do not interfere with the dynamics of the cascade mechanism.

Although it may not be practical to account for the exact relationship between time and quality for every task *i*, some of its attributes can be specified as:

1. Quality increases monotonically as the cumulative resource that drives it $\left({\hat{T}}_{i}\right)$ also increases.
2. If the task receives exactly its planned resource (i.e. ${\hat{T}}_{i}=1$), it will achieve an *almost* perfect quality of completion i.e. *q*(1) = 0.995. This is because its quality can always increase (even by an infinitesimally small increment) if the resource expenditure also increases.
3. A task will have a quality of zero unless some resource has been spent i.e. *q*(0) = 0.

**Fig 4:**
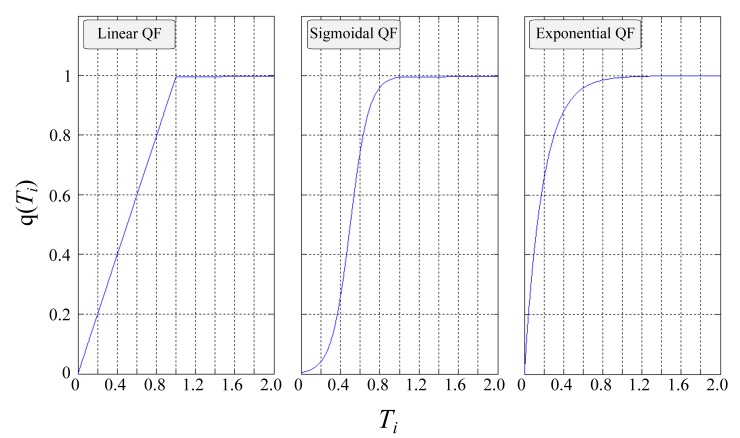
Quality function (QF), showing the relationship between time and completed quality. From left to right, the Linear, Sigmoidal and Exponential QF.

Three distinct functions have been specified—a linear, sigmoidal and exponential relationship–see Fig 4 (see S2 Text for mathematical definitions). Each of these quality functions (QF) captures a number of distinct features. For example, the sigmoidal function (informally knows as the “s-curve”) captures the law of diminishing returns i.e. the closer you reach to your target, the greater the resource expenditure required, a behaviour regularly used to describe numerous process within the domain of PM [1].

### Extrinsic Nodal Characteristics

A number of key network properties are expected to significantly affect the way in which the cascading process unravels. Network structure has been shown to be one such aspect [14, 43–45]; temporal features being another [46–48]. As a consequence, some nodes will have an increased capacity to fuel such cascades i.e. have an increased spreading power $\left(C_{i}^{\text{SP}}\right)$. At the same time, others are expected to be increasingly sensitive $\left(C_{i}^{\text{S}}\right)$ in being affected by such cascades. Consequently, and within this section, both $C_{i}^{\text{SP}\left(\text{m}\right)}$ and $C_{i}^{\text{S}\left(\text{m}\right)}$ will be formally defined by using topological and temporal aspects of the AON network—*m* will denote each aspect used, where *m* = {topo, temp, slack}—see Table 1.

**Table 1:** Overview of extrinsic nodal characteristics.

	Spreading Power, $C_{i}^{\text{SP}}$	Sensitivity, $C_{i}^{\text{S}}$
**Topological Aspects**	$C_{i}^{\text{SP}\left(\text{topo}\right)}$—based on the number of ways node *i* can reach node *j*, weighted over the distance that it has to traverse (Eq 3).	$C_{j}^{\text{S}\left(\text{topo}\right)}$—based on the number of predecessors node *j* has (Eq 7)
**Temporal Aspects**	$C_{i}^{\text{SP}\left(\text{temp}\right)}$—A ratio between the duration of node *i* in relation to the overall duration of the project (Eq 4).	$C_{j}^{\text{S}\left(\text{temp}\right)}$—The inverse ratio between the overall project duration and the duration of node *j* (Eq 8).
		$C_{j}^{\text{S}\left(\text{slack}\right)}$—based on the slack time between node *i* and *j* (Eq 9).

#### Spreading Power for task *i*

The fundamental condition for enabling a cascading process is the existence of at least one link between node *i* and *j*. However, there may be a number of paths in which node *i* can reach node *j*—the greater the number of paths, the more likely it is for node *i* affect node *j*. Importantly, not all paths can be deemed to be of the same importance—longer paths play a lesser role as they are less likely to be traversed [49]. As such, the number of hops needed for node *i* to reach node *j* can be used as weighting for each such path. In other words, the greater the number of hops required for node *i* to reach node *j*, the lower the contribution of that path will be, in terms of a nodes’ spreading power. By summing the number of these weighted paths, a measure of effectiveness in terms of pairwise interactions can be defined i.e. how effective node *i* can reach node *j*, over all possible paths [50, 51]. Let us refer to this quantity as the Pairwise Embeddedness Coefficient (*PEC*) between node *i* and *j*. This quantity can be computed by using the so-called adjacency matrix, defined as:

$$A\left(i,j\right)=\{\begin{array}{c} 1\;\text{if there is a link between}\;i\;\text{and}\;j\\ 0\;\text{otherwise} \end{array}$$

**Fig 5:**
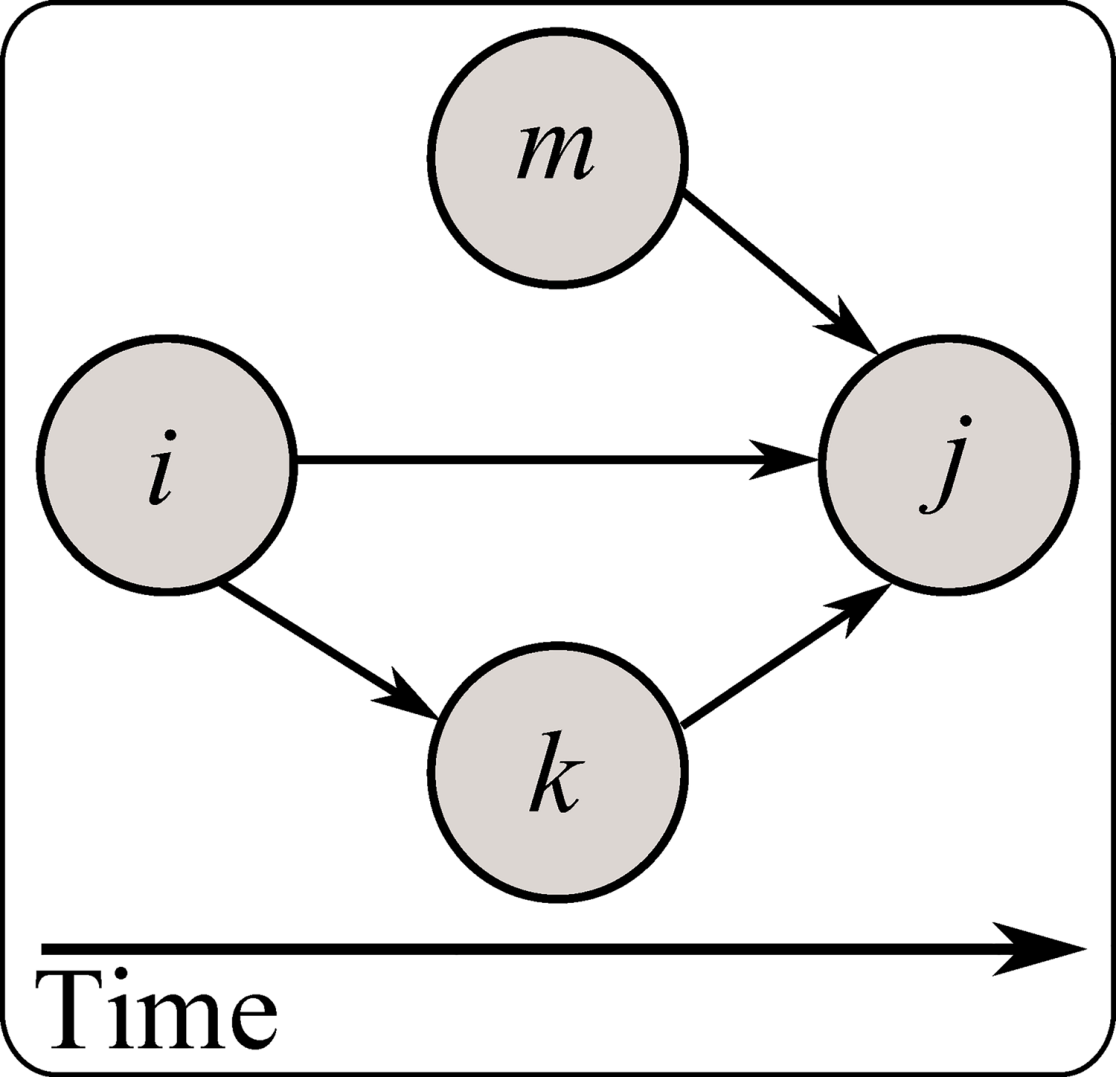
Trivial AON network illustrating the relevance of *PEC* in the context of a cascading process. Node *i* can affect node *j* directly, and indirectly, through node *k*. As such, it has an increased influence on node j, a result of increased network embeddedness, reflected by a high *PEC* value.

A fundamental property of the adjacency matrix is that as it is raised in power *k*, the entry **A** ^***k***^(*i*, *j*) will provide the total number of paths between *i* and *j* of length *k*. This operation can be used to compute *PEC* between node *i* and *j*:

$$\text{Pairwise Embeddedness Coefficient},{PEC}_{i,j}=\sum_{k=1}^{n}\frac{A^{k}\left(i,j\right)}{k}$$ (2)

where *n* is the total number of nodes, *k* is the length of the path between *i* and *j*. As a trivial example, consider Fig 5. One could argue that node *m*, *i* and *k* have equal influence on node *j*, and this may be so. However, it is clear that node *i* can influence node *j* directly but also indirectly through node *k*. Consequently, node *i* has a stronger pairwise interaction (i.e. *PEC*_*i*,*j*_ > *PEC*_*m*,*j*_ = *PEC*_*k*,*j*_) a direct result of its increased network embeddedness.

Consequently, if node *i* has a high *PEC* value with respect to an increased number of nodes, it implies that it is well embedded within the network and thus, well positioned to affect a great number of nodes. This quantity can be calculated by summing *PEC*_*i*,*j*_ over all reachable nodes *j*:

$$C_{i}^{SP\left(\text{topo}\right)}=\sum_{i\neq j}PEC_{i,j}=\sum_{i\neq j}\sum_{k=1}^{n}\frac{A^{k}\left(i,j\right)}{k}$$ (3)

In terms of the temporal aspect of $C_{i}^{\text{SP}}$, task duration is deemed to be of relevance. Specifically, and in the context of PM, the capacity of a task to influence its succeeding task is expected to be a function of how much time the latter is expected to consume, over the entire project duration. The greater this quantity is the greater the influence of the task upon its successors–in other words:

$$C_{i}^{\text{SP}\left(\text{temp}\right)}=\frac{T_{i}^{\text{end}}-T_{i}^{\text{start}}}{T_{N}^{\text{end}}-T_{1}^{\text{start}}}$$ (4)

where task index 1 and *N* represent the first and last task of the project respectively. Note that the overall spreading power is necessarily zero if any of its two constituent aspects is also zero i.e. a task with a given duration and/or out-degree of zero. It can be defined as:

$$C_{i}^{\text{SP}}=C_{i}^{\text{SP}\left(\text{topo}\right)}\times C_{i}^{\text{SP}\left(\text{temp}\right)}$$ (5)

Note that since *q*(*i*) ∈ [0, 1], $C_{i}^{\text{SP}}$ also needs to be normalised within that range:

$${\overset{´}{C}}_{i}^{\text{SP}}=\frac{C_{i}^{\text{SP}}-\text{min}\;C^{\text{SP}}}{\text{max}\;C^{\text{SP}}-\text{min}\;C^{\text{SP}}}\;\text{where}\;{\overset{´}{C}}_{i}^{\text{SP}}\in \left[0,1\right]$$ (6)

#### Failure Sensitivity for task j

As links represent functional dependencies, by increasing the number of predecessor tasks to any given task, an increase in the probability of failure is to be expected—there are simply more things that can go wrong. This topological aspect can be introduced by accounting for the in-degree of node *j*:

$$C_{j}^{\text{S}\left(\text{topo}\right)}=k_{j}^{\text{in}}=\sum_{i=1}^{n}\;A\left(i,j\right)$$ (7)

The temporal aspect of a task’s sensitivity will be a function of two distinct aspects–tasks duration $\left(C_{j}^{\text{S}\left(\text{temp}\right)}\right)$ and the time between the delivery of task *i* and the initiation of task $j\;\left(C_{j}^{\text{S}\left(\text{slack}\right)}\right)$. It is worth noting that although the latter is traditionally used within PM practice (i.e. task free float [52]), it has been shown to affect the operation of various other complex systems [53–56].

**Fig 6:**
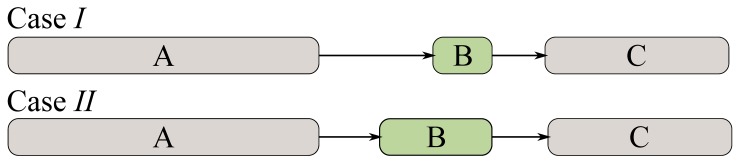
Example of two task sequences (Case *I* and *II*) with same overall duration but different individual duration. Under Case *I*, task B has a smaller duration than its counterpart in Case *II*. Consequently, the inter-event time between task A and B is larger in Case *I* than its counterpart in Case *II*.

Focusing on the influence of task duration, the exact opposite effect of the one introduced in $C_{i}^{\text{SP}\left(\text{temp}\right)}$ is to be expected. Consider the trivial example shown in Fig 6 –in which the duration of Task B is varied. If a risk materializes during the undertaking of Task A, Case *I* is ill prepared to cope against it, compared with Case *II*. This is because Task B has a smaller capacity (i.e. duration) to absorb this external shock and hence, have a reduced ability to add extra working hours to mitigate against it. In other words, a shorter task is expected to be increasingly prone to change as the opportunity to increase working hours within the actual time allocated for it, are less. Mathematically, it can be defined as:

$$C_{j}^{\text{S}\left(\text{temp}\right)}=\frac{T_{N}^{\text{end}}-T_{1}^{\text{start}}}{T_{j}^{\text{end}}-T_{j}^{\text{start}}}$$ (8)

With respect to the time between two consequent tasks, and its effect on node sensitivity, consider Fig 6 in which $T_{B}^{\text{start}}-T_{A}^{\text{end}}$, under Case *I*, is greater than the one in Case *II*. With respect to Task B, if Task A experiences a failure, Case *II* is arguably a worse scenario than Case *I* (assuming all other factors remain constants) as there is less time to react before Task B begins.

To quantify this effect, a similar approach as the one used for computing $C_{i}^{\text{SP}\left(\text{topo}\right)}$ is used, in which the number of hops between nodes is simply replaced by the time difference between two consequent tasks. Note that $C_{j}^{\text{S}\left(\text{temp}\right)}$ is bound to scale with $k_{j}^{\text{in}}$, though this does not necessarily imply that node’s *j* sensitivity has increased, from a temporal point of view. It is rather an artefact induced by node’s *j* topology—this effect can be eliminated by normalising the sum with the in-degree of node *j*:

$$C_{j}^{\text{S}\left(\text{slack}\right)}=\frac{T_{N}^{\text{end}}-T_{1}^{\text{start}}}{\text{max}\left(1,\frac{1}{k_{j}^{\text{in}}}\sum_{i\in I}\left(T_{i}^{\text{end}}-T_{j}^{\text{start}}\right)\right)}$$ (9)

where $k_{j}^{\text{in}}=\sum_{i=1}^{n}A\left(i,j\right),$ *I* is the set of nodes that directly connects to *j* i.e. **A**(*i*, *j*) = 1 and the max function ensures that $C_{j}^{\text{S}\left(\text{slack}\right)}$ has a lower bound.

The overall sensitivity of task *j* is expected to be zero if either of $C_{\text{j}}^{\text{S}\left(\text{topo}\right)},\;C_{\text{j}}^{\text{S}\left(\text{temp}\right)}$ or $C_{\text{j}}^{\text{S}\left(\text{slack}\right)}$ are zero. Hence, a combined metric $C_{\text{j}}^{\text{S}}$ is defined:

$$C_{j}^{\text{S}}=C_{j}^{\text{S}\left(\text{topo}\right)}\times C_{j}^{\text{S}\left(\text{temp}\right)}\;\times C_{j}^{\text{S}\left(\text{slack}\right)}$$ (10)

As with $C_{i}^{\text{SP}}$, $C_{j}^{\text{S}}$ is also normalised in a similar fashion, obtaining ${\overset{´}{C}}_{j}^{\text{S}}\in \left[0,1\right]$. Finally, note that all three metric, and therefore the result metric $C_{j}^{\text{S}}$, are unitless.

### Cascade Process

A threshold value lies at the core of the cascade process, serving as the tipping point of failure propagation—it can be interpreted as the capacity of node *i* to perform. Consequently, it needs to account for essential contextual information. As such, every node *j* is assigned an initial failure threshold, based on its quality of completion (Eq 11, second term on RHS). Subsequently, every node *i* is artificially failed (i.e. its state is switched from “non-affected” to “affected”) and the state of all of its neighbouring nodes *j* is assessed. The amount of stress that they receive will be a function of their own sensitivity $\left({\overset{´}{C}}_{j}^{\text{S}}\right)$, along with the efficiency at which node *i* can convey this stress i.e. its spreading power $\left({\overset{´}{C}}_{i}^{\text{SP}}\right)$—as captured by the first and second term in the LHS of Eq 11 respectively. If the magnitude of this stress is greater than a given percentage of its initial capacity, then node *j* fails is considered and has the opportunity to fuel the cascade further by affecting its own neighbours. Note that the state of every node *j* is defined as *s*_*j*_ ∈ [0, 1], corresponding to “non-affected” or “affected” respectively. Finally, parameter *α* provides the means of devising a spectrum of conditions, where the magnitude of the failure threshold can be controlled–it does so by acting as a scaling factor. Consequently, the higher *α* is, the harder it is for node *j* to be affected. Naturally, the largest cascade is expected to be observed at *α* = 0 while the smallest one should arise at *α* = 200.

The following description details the algorithm that delivers the dynamics (see S3 Text, and S1 Fig, for a worked example):

### Cascade Model Algorithm

*1. Model parameterisation:*

- Set model parameter: *α* ∈ [1, 200]

- Define a quality function: *QF*.

- Define which node is artificially failed at the start of the simulation: *k*

*2. Model initialisation:*

- Draw task fluctuations $\beta_{i}^{\text{pdf}}$ from a statistical distribution.

- Set $q_{i}\;=\;QF\left[\left(T_{i}^{\text{plan}}+\beta_{i}^{\text{pdf}}\right)/\left(T_{i}^{\text{plan}}\right)\right]$ for all *i*

- Define initial node states at time *t* = 0: $s_{i}\left(0\right)=\{\begin{array}{l} 1,\;if\;i==k\\ \;0,\;otherwise \end{array}$

*3. Model dynamics:*

- Iterate simulated time *t* = 1, 2, …, until no more change happens:

    - For all ‘affected’ nodes *i* (i.e. nodes where *s*_*i*_ = = 1):

        - For all successor nodes *j* of node i:

$$s_{j}\left(t\right)=\{\begin{array}{l} 1,\;\text{if}\;C_{j}^{\text{S}}+C_{i}^{\text{SP}}\geq \frac{\alpha }{100}\times q_{j}\\ \;s_{j}\left(t-1\right),\;\text{otherwise} \end{array}$$ (11)

*4. Model outcome:*

- The number of affected nodes: $\frac{1}{n}\sum_{i=1}^{n}\;{\text{s}}_{i}$

The condition controlling the dynamics (Eq 11) is driven by the magnitude of change experienced by each task. As such, it suggests that an activity has a given capacity to tolerate fluctuations when things do not go as planned, in terms of resource allocation. This view differs from typical cascade models, where the state of a node changes according to an *absolute* threshold value (either drawn from empirical estimates, e.g. [57], or purely arbitrary values e.g. [45]). In the case of projects, the application of absolute thresholds will not apply due to the inherently large variation in the nature of tasks, where no absolute value can provide for a sensible threshold for all possible types of tasks. In response, Eq 11 introduces a *relative* threshold, shifting the condition of failure to the magnitude of the relative change rather than an absolute value. By doing so, it assumes that tasks will not necessarily affect their successors in light of their own poor quality, but rather on their inability to cope with large changes. In other words, it is more likely for a poorly completed task to still be delivered, unlocking its successors, when compared to one that has a relatively higher level of quality but has sustained extensive changes.

## Results

### Initial Setup

This model has three main parameters that can be controlled–the pdf of which $\beta_{i}^{\text{pdf}}$ is drawn from (Fig 2); the QF used to define the relationship between resource and quality (Fig 3) and control parameter *α*. The first captures the volatility of the environment in which the project takes place; the second relates to the nature of the task itself; the third provides a global variable that increases the difficulty of which a cascade can progress. In step with the scope of this work, only the latter will be varied–both the pdf and QF used will be set as constants. Specifically, the pdf will be set to Normal, representing a stable environment in which the probability of obtaining increasingly large $\beta_{i}^{\text{pdf}}$ values decreases exponentially. With respect to used QF, the Sigmoidal variant will be chosen due to its widespread use in describing various PM activities; the entire set of results can be found in S3 Table. Nevertheless, and for the sake of completeness, results of the Linear and Exponential QF are also included–see S2 Fig

After this initial setup, node *i*, ∀*i* ∈ *N*, is forced to fail and its impact captured by enumerating the size of the triggered cascade—this process is repeated across a range of *α* values, ∀*α* ∈ [0, 200]. It is worth noting that the mere observation of any significant cascades, under this set-up, highlights the fact that *no* great exogenous factor is required for global catastrophes to occur. In other words, the role of the environment in the emergence of cascades is minimal, limited to simply triggering a single, local failure. Once this local impact has been registered, the mechanism responsible for driving the cascade is entirely self-contained, emphasizing the importance of the underlying AON network.

### Model Output

**Fig 7:**
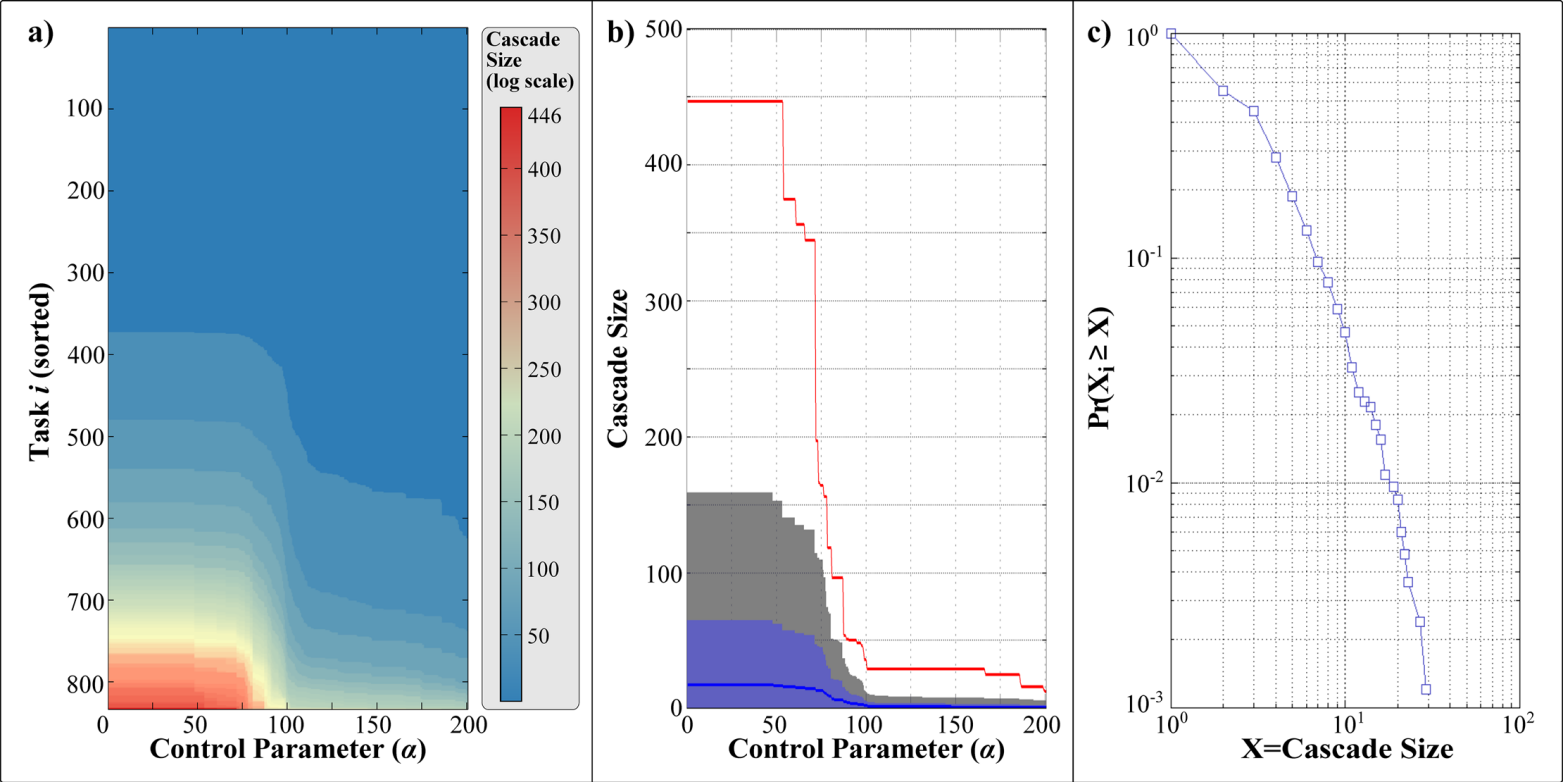
Model results. a) Size of cascade (colour, log scale) as each node *i* (y-axis) is purposefully failed across a range of *α*, ∀*α* ∈ [0, 200] (x-axis). b) Captures the aggregated state in which the maximum/mean cascade size is mapped (y-axis) across the entire spectrum of *α* (x-axis). Finally, c) captures the cumulative probability distribution of cascade sizes, at *α* = 0. Note the log-log scale used.

Interestingly, a large percentage of nodes are incapable of inducing any form of cascade–this is clearly marked by the abundance of blue hue at *α* = 0—see Fig 7A. Naturally, this percentage increases as *α* also increases. In the domain of *α* ∈ [0, ≅ 50], the capacity of the nodes to induce any given cascade shows no change, noted by the constant gradient across all colours. In other words, the impact of having *no* resistance to failure (the direct result of setting *α* = 0) continues up to *α* ≅ 50 (Fig 7A, blue hue). At this point, the size of the maximum cascade is 446 –see Fig 7B. As *α* continues to increase, cascade sizes drop, with the rate of decrease in size growing significantly once *α* grows above approximately 75. Interestingly, the rate of decrease appears to be proportional to the maximum impact of each node i.e. large cascades diminish faster than smaller ones. Once *α* reaches 100, the maximum cascade size stabilises to 29, and remains relatively constant, despite occasional reductions which result to a final maximum cascade size of 13, at *α* = 200—see Fig 7B.

It should be noted that the difference between the largest and the average cascade is substantial, and remains so across all *α*, ranging from 5.10 and up to 13.21 standard deviations. Clearly, such variance would not be possible under a Gaussian distribution (or any other function with an exponential tail). Consider Fig 7C, as it presents the cumulative probability distribution of all obtained cascade sizes, averaged across *α*. The obtained straight line under a log-log plot highlights that the distribution resembles a power law, in which *f*(*x*)∼*x* ^*ε*^ where *ε* is the scaling exponent–in this case *ε* = 1.57. Since *ε* < 2, the distribution has a theoretically infinite mean and variance; in reality it is bound to be capped by the finite size of the system. In other words, the size of the largest possible cascades is capped by the number of nodes that can be reached by any single node. Analogous behaviour has been observed in numerous other interactive systems and has been linked to the emergence of complexity–see Section 4 for a discussion on its implications in the context of PM.

### Task Characteristics as Proxies

Seeking to rank tasks based on the cascade size triggered by their failure poses a methodological question–are extensive simulations necessary or could task characteristics serve as proxies? Importantly, these task characteristics need to be widely captured and easily identified via standard practice–task duration; count of task successors and start date are used and assessed within this sub-section.

**Fig 8:**
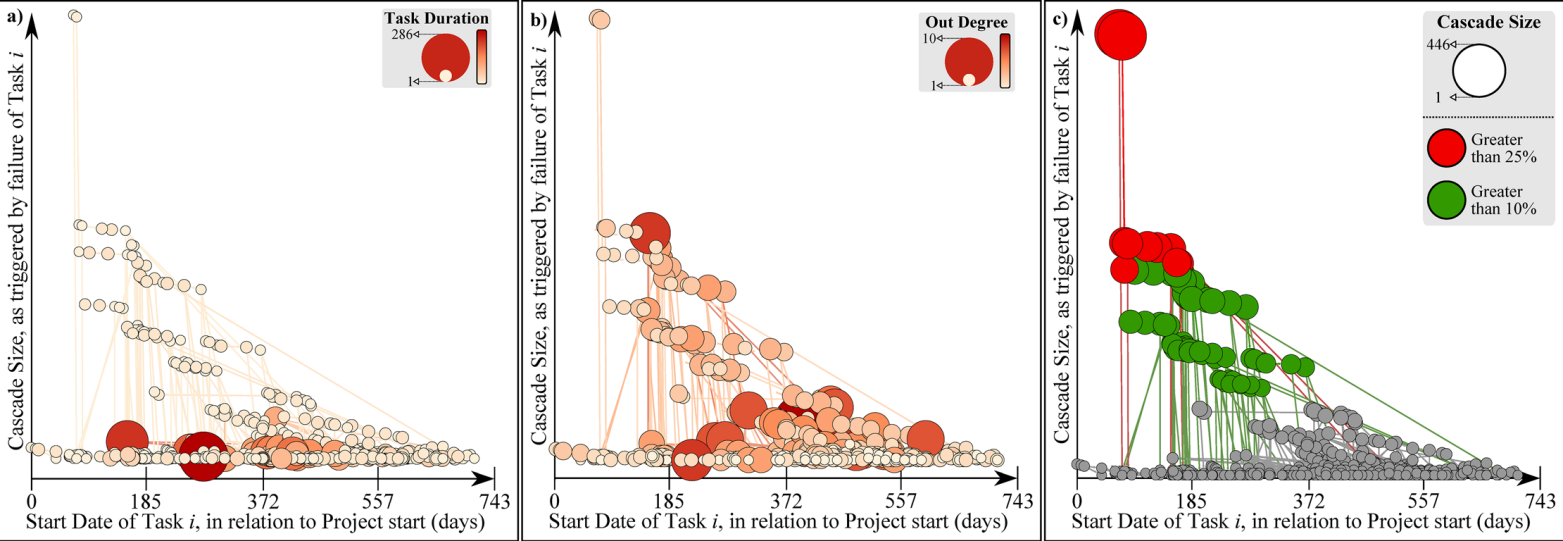
Task Characteristics as proxies for capacity of task to trigger cascades, under *α* = 0. Tasks are mapped according to their start date (x-axis) and in terms of the impact that their failure will have, in terms of cascade size (y-axis). In a) task duration (node size and colour) is assessed as a potential proxy; in b) the out-degree. c) Tasks capable of affecting at least 25% of the total number of tasks are shown (red); at least 10% are also shown (green).

With respect to task duration (Fig 8A), it is clear that the majority of long-duration tasks occur mid-point of the project–however those tasks are not necessarily the ones that cause the greatest cascades. In fact, the two most critical tasks (Task 350 and 351) have duration of 3 and 4 days respectively (compared to the maximum task duration of 286 days), yet their failure can affect a total of 446 and 444 tasks respectively (Table 2). On the other hand, failure of the two longest tasks (Task 719 and 199) will affect a total of 2 and 17 tasks respectively, having a significantly smaller impact. Similar trends are noted throughout the entirety of nodes–see Table 2 for an example on the 5 top impacting (left section) and longest tasks (right section) respectively. In terms of the successors of each task, this can be quantified as the node’s out-degree–see Fig 8B. In terms of number of successors and cascade size, no significant correlation has also been noted–see Fig 8B. As an example, consider Task 3 and 42, with 10 and 8 successors tasks respectively (two highest ranking)–their failure will impact 40 and 14 tasks respectively. Interestingly, this low correlation between node out-degree and failure cascade size also extends to node betweenness. Specifically, the Pearson Correlation coefficient between cascade sizes and node between is 0.6370 (*p*-value<0.0001). For reference, the Pearson Correlation coefficient between out-degree and failure cascade sizes is 0.3359 (*p*-value<0.0001).

**Table 2:** Task characteristics for the 5 highest ranking tasks, in terms of cascade size (a) and task duration (b).

a) Tasks, ranked based on cascade size	Cascade Size	Task Duration	Task Start Date	b) Tasks, ranked based on task duration	Task Duration	Cascade Size	Task Start Date
**1^st^ Task**	446	3	+70	**1^st^ Task**	286	2	+285
**2^nd^ Task**	444	4	+75	**2^nd^ Task**	234	17	+159
**3^rd^ Task**	235	3	+79	**3^rd^ Task**	186	7	+264
**4^th^ Task**	234	1	+84	**4^th^ Task**	170	5	+488
**5^th^ Task**	231	12	+117	**5^th^ Task**	169	6	+390

a) Top 5 tasks, ranked on the maximum cascade size they can trigger, along with their respective proxies such as task duration and their start date relative to the start of the project

b) Top 5 tasks, ranked on their task duration, along with the maximum cascade size they can trigger and their start date.

Finally, note that the entirety of nodes that can significantly impact the project is scheduled at the 1^st^ half of the project. Specifically, tasks that significantly impact the project (i.e. affect at least 25% of all tasks) take place at the early stages of the project, being limited to the 1^st^ quarter of the project’s duration. The same applies for tasks capable of affecting at least 10% of all tasks, though they are spread more evenly throughout the 1^st^ half of the project–see Fig 8C.

## Discussion

**Fig 9:**
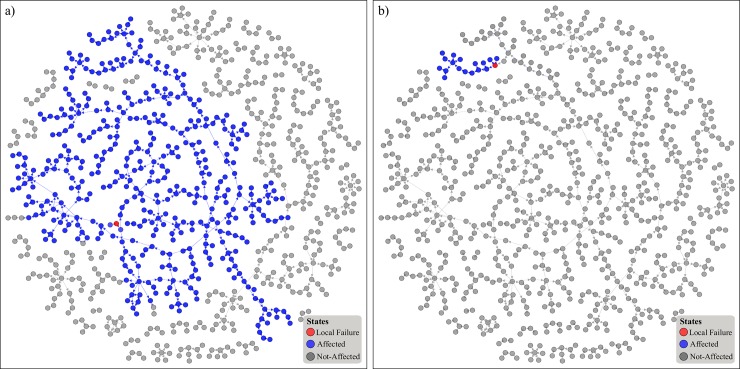
Visual example on the progression of the largest and average cascade, under *α* = 0. a) The source of the largest noted cascade (red), along with the subsequently failed nodes (blue). b) Same as a), but capturing a typical cascade.

This work is based upon a self-contained model that captures the emergence of large catastrophes (i.e. no external input is required to sustain the cascading process). By doing so, a number of important insights can be drawn. Firstly, it suggests that large catastrophes *do not* necessary require a great exogenous force to emerge–in fact, a single local failure can be sufficient in taking out a large portion of the project. This is of great practical relevance as it challenges a key aspect of project management–the impact assessment of a risk, in an a *priory* fashion [1, 58]. As such, the effectiveness and efficiency of the subsequent mitigation action may be mismatched, resulting in an escalation of failures or unnecessary resource expenditure. Quantifying this aspect, along with tracing it back to the tasks responsible for it, enables proactive project (and risk) management, striving for increased project robustness. In response, Fig 7A captures this aspect at a global level, summarising the cascade size induced by failing each node *i*. Equally, Fig 9A and 9B provides a visual example of two nodes of which their failure can induce the largest and an average cascade respectively. Such means of analysis enable project managers to quantitatively and objectively assess the criticality of each task and prioritise resource allocation and mitigation planning accordingly. Equally, one could aggregate the criticality of all tasks and devise a high-level profile (e.g. Fig 7), enabling project-level comparisons within a portfolio. By doing so, projects capable of sustaining larger cascades, assuming a local failure does indeed occur, can be identified and managed accordingly. It is worth noting that the majority of tasks has a limited capacity of triggering a cascade–in fact 391 tasks induce *no* cascade, with an average cascade of 17.29 at the worst case scenario (i.e. when *α* = 0). Hence, it is rather surprising that the maximum cascade deviates from the mean to such a great extent (nearly 10 standard deviations). Evidence of this sort highlight the extreme asymmetry in the capacity of tasks to impact the project, emphasizing that no single management approach can be applied to all of them. Some tasks are distinctly different in terms of their capacity to impact the project and should be treated as such.

By acknowledging this asymmetry, it is worth exploring whether crude task characteristics can provide hints of task capacity, or whether simulation-based approaches, as the one described within this work, are necessary. Trivially, one would expect that tasks scheduled at the early stages of the project would be more critical as they have a larger pool of potential tasks to affect. With reference to Fig 8C–this is indeed the case, evident by the far left location of all red (and some green) nodes on the x-axis. However, a number of influential tasks (i.e. remaining green nodes) can also be seen at the latter stage of the project. In a PM context, this implies that even if tasks are performing well at the initial stages of a project, this observation cannot serve as a predictive proxy of future project performance, as some tasks that are yet to be completed still have the capacity to affect a relatively large percentage of all tasks (greater than 10%). With respect to Fig 8A and 8B, it is clear than neither the number of task successors nor the duration can adequately serve as proxies for the even qualitatively assessing task criticality. In fact, further analysis indicates that another widely used metric (node betweenness) perform poorly in predicting the influence of all tasks, with respect to the size of a failure cascade that they can induce. This view is in line with the critical work of Lawyer [59], where he argues that the utility of such metrics in limited due to their lack of accuracy. Nonetheless, adopting a network perspective does provide some intuitive insight over traditional approaches–see Fig 9. Specifically, the task responsible for the largest cascade (Fig 9A) appears to be well embedded within the system. In contrast, a task capable of triggering an average cascade (Fig 9B) appears to be on the outskirts of the structure. Project managers can use this insight in order to quickly identify potential candidate tasks that may be critical while avoiding extensive simulation. Nevertheless, it is clear that if increased precision and rigor are required, approaches like the one described within this work have a lot to offer.

From a practical perspective, increasing the resilience of a project is bound to be a challenging task, due to limitations in amending the underlying network structure. Specifically, as links represent functional interdependences between tasks, it is hard to rewire them without imposing significant changes on a project’s outcome. One way of getting around this limitation is to decouple the successor tasks of critical tasks by assigning them to different contractor(s). By doing so, the failure of a critical task will have a limited impact on its subsequent tasks, assuming the operation of such contractors is independent. Influencing temporal aspects of a project can further increase the resilience of a project. Preliminary work indicates that by changing the IET distribution from being heavily-skewed to a normal one (in effect, minimising the variance) improves the resilience of the entire project–this insight is consistent with results reported in [47]. As such, projects described by temporal homogeneity are expected to be more robust. Nonetheless, further work is needed to develop means of overcoming various restrictions (e.g. resource availability, cash-flow) before being able to change the IET of all tasks.

On a wider scope, this work also draws certain analogies between projects and *natural* systems. Specifically, the fact that small failures can have an extensive impact on the overall system, along with the noted power law that describes the cascade size distribution, have been noted in numerous other domains, ranging from geology to economics. Self-Organised Criticality (SOC) is a general theory, first proposed by Bak, Tang [60], and has formed the basis of devising general principles around the emergence of complexity within those systems. Such generality is mostly absent from management science as the widely-voiced proposition of each project being unique fosters the development of case-specific knowledge. Hence, by highlighting aspects of SOC within projects, parallels can be drawn within the theory that describes the behaviour of these natural systems. By doing so, one could envision the emergence of wider principles that can be applied to an increased number of projects, contradicting the very notion of a project being a “*unique* endeavour” but rather being a system of which its complexity could equally be compared with other natural systems.

Unfortunately, the distinct character of the AON networks (compared to other widely used synthetic networks) poses a significant restriction on testing the universality of the results presented herein. As such, further exploration of the analogy between project behaviour (and the ability to construct a widely applicable theory) and natural systems are a challenging task, as empirical data remains the sole source of AON networks. Working towards clarifying this aspect, the authors have been involved in a larger study (involving a total of 8 AON networks) where qualitative similar results were obtained (draft manuscript available upon request). Consequently, the capacity of projects to sustain disproportionately large cascading failures, along with the challenged notion project uniqueness (and the subsequent implications on wider-project theory) is not outlier behaviour but rather a wider phenomenon worth further exploration due to its important practical implications.

## Conclusion

A project can be seen as a large interactive system [38, 61] in which complexity emerges at a number of dimensions [8, 62]. As a direct result of this complexity, a number of great failures have recently been noted [11–13]. This work focuses on a specific failure mode that is enabled by such structural complexity [62]–the capacity of a local failure to emerge as a global catastrophe via a cascading process [18, 19]. To do so, a set of mechanisms were constructed, based on a general model (i.e. the threshold model) that was suitably modified to reflect context specific aspects such as quality of task completion. This process was modelled against an empirical AON network.

In doing so, nodes that had a capacity to induce disproportionately large cascades were identified, along with illustrating the fact that a great number of nodes were incapable of sparking *any* cascades (Fig 7A and 7B). Similarly, it was shown that crude, project management orientated information such as task duration, number of successors and task start date provide no significant insight in terms of the task capacity to trigger a cascade (Fig 8)–similar insufficiencies were under various network-based metrics such as node degree and node betweenness. Hence, analysis of this sort is of significant practical utility as it provides important, practical information while using a widely used data-structure. By doing so, a number of proactive risk mitigation techniques can be applied by decision makers (e.g. decoupling critical tasks, narrowing the variance of the IET distribution) in order to improve the robustness of a project. Finally, parallels with SOC, as encountered in numerous natural systems, were drawn (e.g. Fig 8C), suggesting commonalities between projects and other complex systems. If that is indeed the case, then here lies an opportunity for transitioning from a case-by-case understanding to developing general principles for project management. This is a widely sought but currently eluding quality of management science, reflecting the generally voiced opinion that every project is unique—such evidence highlights that the contrary may also be true.

## Supporting Information

S1 Dataset Original Gantt chart (task description in Greek). (MPP)

S1 Fig Simple AON used to construct the worked example, as found in S2 Text. (TIF)

S2 Fig Results under the case of the Linear QF and Exponential QF. (TIF)

S1 Table Edge list capturing the structure of the AON network used. Note that the edge list refers to task indices and not their actual ID.
(XLSX)

S2 Table Table containing task information, as extracted from the actual Gantt Chart. Information includes the task index, task ID, its duration (in days) and their start date, in relation to the start of the project.
(XLSX)

S3 Table Raw result output of the analysis. This table includes the raw output of the model, under the case of the Sigmoidal QF. Each row represents the task index, with each column reflecting the *α* value used in Eq 11.
(XLSX)

S1 Text Process for converting a Gantt Chart to an AON network. (DOCX)

S2 Text Mathematical definitions of the 3 QF used, as seen in Fig 4. (DOCX)

S3 Text A worked example of the cascade model, on a simple AON network. (DOCX)
