# Supplementary figures and images for: How Robust Is Your Project? From Local Failures to Global Catastrophes: A Complex Networks Approach to Project Systemic Risk
Source: PLoS One. 2015 Nov 25;10(11):e0142469. doi: 10.1371/journal.pone.0142469 (PMC4659599; doi:10.1371/journal.pone.0142469)

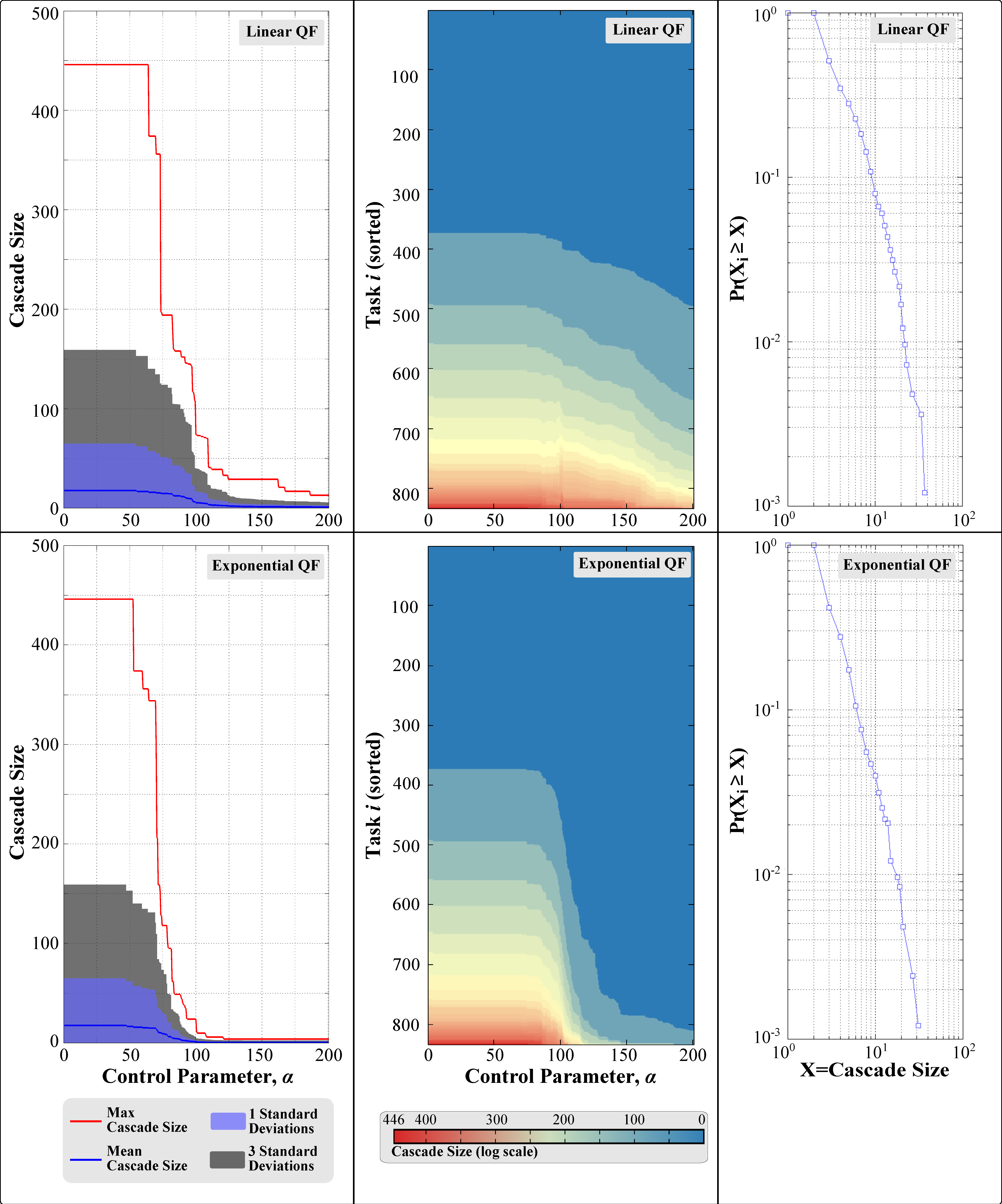

Supplement: S2 Fig — (TIF) [file pone.0142469.s003.tif]
