# Supplementary material for: How Robust Is Your Project? From Local Failures to Global Catastrophes: A Complex Networks Approach to Project Systemic Risk
Source: PLoS One. 2015 Nov 25;10(11):e0142469. doi: 10.1371/journal.pone.0142469 (PMC4659599; doi:10.1371/journal.pone.0142469)
Supplement: S1 Text — (DOCX) [file pone.0142469.s007.docx]

**S4 Text**

**Process in converting a Gantt chart into an AON network**

1. Open Gantt chart in MS Project (or equivalent)
2. Ensure the outline is set to the maximum level, to ensure the entirety of tasks are visible
3. Use the built in function to save the task information as an MS Excel file (.xlsx), ensuring that the following column are includes
   - Task ID
   - Duration
   - Start Date
   - Successor (Tasks)
4. Open file in MS Excel (or import into a suitable programming suite e.g. Matlab) and clean up file to remove Tasks that serve as heading in the Gantt Chart. These are Tasks that have both Zero links (i.e. no successors and do not belong to the successor set of any other task) *or* zero duration
5. The remaining Successor list is essentially an edge list and can be used to compute the full adjacency matrix of the network
