# Supplementary material for: How Robust Is Your Project? From Local Failures to Global Catastrophes: A Complex Networks Approach to Project Systemic Risk
Source: PLoS One. 2015 Nov 25;10(11):e0142469. doi: 10.1371/journal.pone.0142469 (PMC4659599; doi:10.1371/journal.pone.0142469)
Supplement: S2 Text — (DOCX) [file pone.0142469.s008.docx]

## **S6 Text**

## **Mathematical definitions for QF**

The Linear QF, as seen in Fig, 4, left subplot, is defined as:

|  | $q^{\mathrm{lin}}\left( \hat{T_{i}} \right)=\left\{ \begin{aligned} \beta\times\hat{T_{i}}, \mathrm{if} \hat{T_{i}}\leq1 \\ \frac{\hat{T_{i}}-0.005}{\hat{T_{i}}}, \mathrm{if} \hat{T_{i}}>1 \end{aligned} \right. \mathrm{where} \beta=q\left( 1 \right)=0.995$ | (1S3) |
| --- | --- | --- |

The Sigmoidal QF, as seen in Fig, 4, centre subplot, is defined as:

|  | $q^{\mathrm{sigm}}\left( \hat{T_{i}} \right)=\frac{1}{1+\exp\left( -\gamma\hat{T_{i}}+\beta\right)}, \mathrm{where} \beta=-\ln\left( \frac{1}{q\left( 1 \right)}-1 \right) \mathrm{and}\gamma=2\beta$ | (2S3) |
| --- | --- | --- |

The Exponential QF, as seen in Fig, 4, right subplot, is defined as:

|  | $q^{\exp}\left( \hat{T_{i}} \right)=1-\frac{1}{\exp\left( \beta\hat{T_{i}} \right)}, where \beta=-ln(1-q\left( 1 \right))$ | (3S3) |
| --- | --- | --- |
