# Supplementary material for: How Robust Is Your Project? From Local Failures to Global Catastrophes: A Complex Networks Approach to Project Systemic Risk
Source: PLoS One. 2015 Nov 25;10(11):e0142469. doi: 10.1371/journal.pone.0142469 (PMC4659599; doi:10.1371/journal.pone.0142469)
Supplement: S3 Text — (DOCX) [file pone.0142469.s009.docx]

**S6 Text**

**Worked example of the cascade process**

With respect to S1 Fig., a trivial AON network is presented, where failure of node 3 has been induced. The effect of this local failure is followed at two distinct cases of the control parameter. Let us focus at$t=2$, as that is the defining point where the differing$\alpha$ values exemplify their impact. In the case of$\alpha=10$, $\theta_{4}^{1}=q_{4}^{\mathrm{sigm}}=0.654$ while the threshold at the second time step has been reduced to 0.204 (i.e.$\theta_{4}^{2}=q_{4}^{\mathrm{sigm}}-\acute{{SP}_{3}}-\acute{S_{4}})$. Thus, $\frac{\theta_{4}^{1}-\theta_{4}^{2}}{\theta_{4}^{1}}=\frac{0.854-0.504}{0.854}=0.410>\frac{\alpha\times\theta_{4}^{1}}{100}=\frac{10\times0.854}{100}=0.085$. As this change is greater than 10%, node 4 fails. However, when α=50, the cascading process would be halted as the induced change is less than 50% of its initial, un-stressed state.
